# Carrier-Based Drug Delivery System for Treatment of Acne

**DOI:** 10.1155/2014/276260

**Published:** 2014-02-09

**Authors:** Amber Vyas, Avinesh Kumar Sonker, Bina Gidwani

**Affiliations:** University Institute of Pharmacy, Pandit Ravishankar Shukla University, Raipur 492 010, India

## Abstract

Approximately 95% of the population suffers at some point in their lifetime from acne vulgaris. Acne is a multifactorial disease of the pilosebaceous unit. This inflammatory skin disorder is most common in adolescents but also affects neonates, prepubescent children, and adults. Topical conventional systems are associated with various side effects. Novel drug delivery systems have been used to reduce the side effect of drugs commonly used in the topical treatment of acne. Topical treatment of acne with active pharmaceutical ingredients (API) makes direct contact with the target site before entering the systemic circulation which reduces the systemic side effect of the parenteral or oral administration of drug. The objective of the present review is to discuss the conventional delivery systems available for acne, their drawbacks, and limitations. The advantages, disadvantages, and outcome of using various carrier-based delivery systems like liposomes, niosomes, solid lipid nanoparticles, and so forth, are explained. This paper emphasizes approaches to overcome the drawbacks and limitations associated with the conventional system and the advances and application that are poised to further enhance the efficacy of topical acne formulations, offering the possibility of simplified dosing regimen that may improve treatment outcomes using novel delivery system.

## 1. Introduction

Approximately 95% of the population suffers at some point in their lifetime from acne vulgaris [1]. Papules, pustules, closed and open comedones, cysts, and scarring may be seen. Having acne can give rise to feelings of embarrassment, loss of self-esteem, and depression, as well as physical symptoms (such as soreness and pain) associated with individual lesions. Acne is well known to respond to hormones, both endogenous and exogenous. It is the most common dermatologic disorder affecting approximately 85% of the teenagers [2, 3] and a chronic inflammatory follicular disorder of the skin, occurring in specialized pilosebaceous units on the face consisting of the follicular canal with its rudimentary hair, and the group of sebaceous glands that surround and open on to the follicle [4–6].

**Figure 1:**
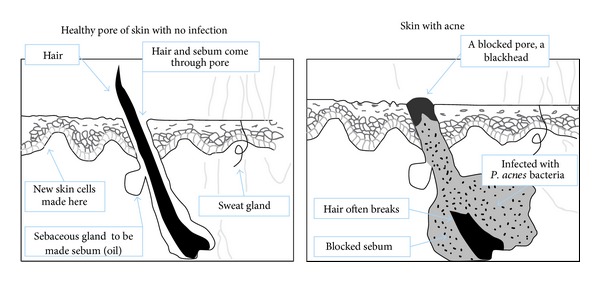
Difference between normal skin and skin with acne.

Acne vulgaris can be defined as the most common skin disease, that results in comedos or severe inflammatory lesions in the face, back, and chest with a large number of sebaceous follicles, and the condition of the disease is associated with the elevated rate of sebum excretion [7]. The pathophysiology of acne includes abnormal proliferation and differentiation of keratinocytes, increased sebum production, hyper proliferation of *Propionibacterium acne*, and inflammatory response initiated by bacterial antigens and cytokines [8–14].  Figure 1 highlights the difference between a normal skin and the skin with acne. In the skin with acne due to the excess production of male hormone androgen and oil producing glands in the face comedone occurs on the face [15]. The closed comedone (whitehead) and ripen comedone (blackhead) are the primary two noninflammatory lesions in acne. These lesions may progress to inflammatory papules and pustules when the contents rupture. Larger, more painful lesions, such as cysts and nodules, may also develop [16]. The application of novel delivery systems to the skin distributes the topical agent gradually, reduces the irritancy of some antiacne drugs, and shows good efficacy [17].

## 2. Status of Acne

**Figure 2:**
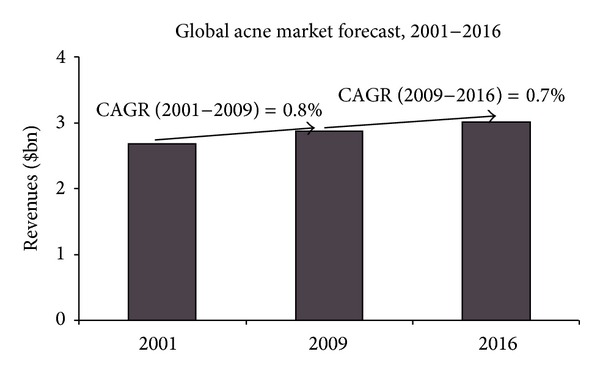
Future forecast of acne.

The severity of acne is rated according to the combined acne severity classification that classifies acne into mild, moderate, and severe based on the number and type of lesions. Tables 1 and 2 enlist the features of different types of acne classified according to their general features and severity [18]. The acne therapeutics market is forecast to show moderate growth in revenues till 2016. The research suggests that the global acne market was worth $2.8 billion in 2009. It is estimated to reach revenues of $3.02 billion by 2016 at a compound annual growth rate (CAGR) of 0.7%.  Figure 2 highlights the global market forecast of acne. The moderate increase in revenues is attributed to overcrowding of the market with generics and the increased acceptance of alternative therapies such as photodynamic therapy and ultraviolet (UV)/blue light therapy. The current market has several products, which act on acne by targeting different etiologic factors involved in the development of acne [19]. Prevalence of acne declines dramatically after the age of 25 to 8%. Acne affects between 40 million and 50 million individuals in the United States [20]. In India the antiacne market was 130 crores, and growing at the rate of 14% annually according to the report of 2009, it would perhaps be now estimated as per the growth standards to somewhere around Rs. 168.94 or Rs. 169 crores in coming years.

## 3. Treatment Strategies Used for Acne

Accordingly, no simple recipe for treatment can be given, and treatment options vary with the stage and intensity of the disease [21–24]. According to the evolution, acne can be classified as mild, moderate, or severe. Topical treatment is the first choice in mild and moderate acne, whereas systemic therapy is used to treat severe and moderate cases [25]. Acne is mainly treated in three different ways.

**Figure 3:**
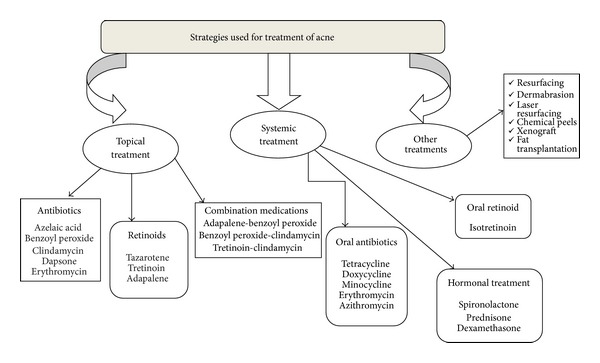
Treatment strategies used for acne.

- Topical therapy: it includes the use of antibiotics, retinoids, and combination medication. Topical acne medications are usually irritating to the skin; more than 40% of acne bacteria are insensitive to oral antibiotics and are associated with possible severe side effects and high cost.
- Systemic treatment includes oral antibiotics, retinoids, and hormonal treatment. Systemic treatment is indicated for the management of moderate and severe acne, acne that is resistant to topical treatment and acne that covers large parts of the body surface.  Table 3 highlights the drugs used in systemic and hormonal treatment of acne.
- Other treatments include those which are not in above two categories like resurfacing, dermabrasion, chemical peels, xenografts, heterograft, autograft and fat transplantation.  Figure 3 enlists a detailed overview of all the strategies used in treatment of acne along with their examples.

## 4. Conventional Delivery System Used in Treatment of Acne 

In earlier days, people all over the world used conventional system for treatment of acne. In the 1950s, antibiotics proved to be effective in treating acne because of the anti-inflammatory effects of tetracycline. Retin A, discovered in the 1960s, was found to fight acne blemishes. Accutane, a form of vitamin A for reducing oil made by skin glands, was introduced as a treatment in the 1980s. Laser treatments for treating acne began in the 1990s and were found to be especially effective for people suffering from nodular and cystic acne. In the 1970s, tretinoin (original Trade Name Retin A) was found to be effective for acne [26]. This preceded the development of oral isotretinoin (sold as Accutane and Roaccutane) in 1980 [27]. Also, some antibiotics like minocycline were used for treatment for acne [28, 29].

From the nineteenth to twentieth century, almost all the treatments of acne were based on conventional system. Conventional available dosage forms/delivery system works by the following four mechanisms, namely, normalizing shedding into the pore to prevent blockage, killing *propionibacterium acnes*, anti-inflammatory effects, and hormonal manipulation [8, 14]. In spite of various available treatments for acne, many patients fail to respond adequately or develop problematic side effects. Most of the conventional available formulations usually produce a high incidence of side effects and symptoms that diminish the patient compliance, compromising the efficacy of the therapy [30–32]. Nevertheless, some of them also lead to skin dryness, peeling and skin irritation, or bacterial resistance [33–37].  Table 4 explains the side effects associated with the conventional formulations used in acne.

To reduce these above mentioned side effects, development of novel carrier-based drug delivery systems came into existence. The application of these novel delivery systems is advantageous to the skin as it distributes the topical agent gradually and in some cases has demonstrated the ability to reduce the irritancy of some antiacne drugs, yet it maintains a better efficacy when compared with conventional formulations [17]. The novel drug delivery systems also have the advantage of penetrating more efficiently into the hair follicles than do nonparticulate systems, such as conventional formulations, so long as the size is selected in an appropriate manner. This provides a high local concentration over a prolonged period [38–40].

## 5. Novel Drug Delivery System Used to Treat Acne

The efficacy of the antiacne topical drugs using novel carrier-based drug delivery system is well established. The local side effects, however, mainly cutaneous irritation, erythema, dryness, peeling, and scaling, remain major problems. The antiacne drug-loaded vesicular and particulate delivery systems (liposomes, polymeric microspheres, and solid lipid nanoparticles) for topical treatment are advantageous compared to conventional available topical delivery system. The encapsulation of antiacne drugs in vesicular and particulate delivery systems represents an innovative and alternative approach for minimizing the side effects and preserving their efficacy.

**Figure 4:**
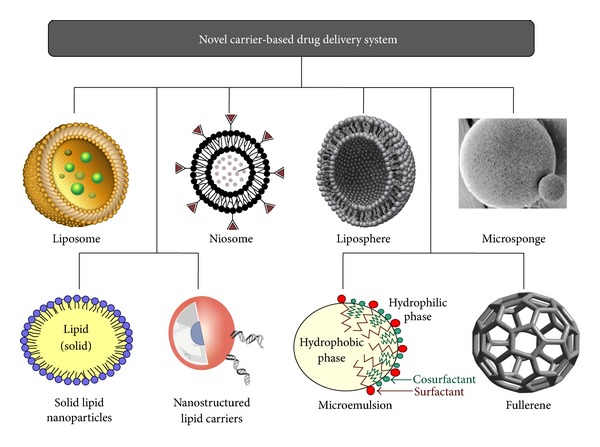
Novel carrier-based drug delivery system for treatment of acne.

Novel drug carriers intended for use in skin diseases are often designed to increase the load ability of APIs and reduce side effect. In dermatotherapy, research on new drug entities and drug delivery systems is focused on frequent diseases often difficult to treat, in particular acne and psoriasis [54]. For severe manifestations, not infrequent highly active APIs, which may also induce major unwanted effects, have to be prescribed for systemic use. Progress in novel drug delivery systems may allow the safer use of these agents by the topical route [55]. The novel carrier systems that are under investigation for application and treatment of acne include liposome, niosome, microsponge, microemulsion, microsphere, SLN, hydrogel, aerosol, fullerenes and so forth [54, 55]. Controlled drug release of these novel carrier-based delivery systems and subsequent biodegradation are essential for developing successful formulations. The drug release mechanism of these systems involves desorption of adsorbed drug, diffusion through the carrier matrix, erosion, and combination of erosion and diffusion method. Along with the numerous advantages, novel vesicular carrier system is associated with some serious disadvantages which restrict their use: drugs passively may lead to low drug loading efficiency and drug leakage in preparation, preservation, and transport *in-vivo*. Also, the major problem of their stability acts as a barrier and limits their use [56]. The novel carrier based delivery systems are discussed with their advantages, limitations, and suitable examples.  Figure 4 shows various carrier-based drug delivery systems used in treatment of acne.

### 5.1. Liposomes

Liposomes are spherical particles composed of one, several, or multiple concentric membranes [57]. They are potent drug delivery systems for treating hair follicle-associated disorders such as acne [58].

#### 5.1.1. Advantage of Liposomal Formulation

After topical application, liposome can improve drug deposition within the skin at the site of action, reduces systemic absorption, and minimizes the side effects thereby providing localized effect [59]. They can target the drug to skin appendages in addition and increase the systemic absorption [60]. They can improve the therapeutic effect of drugs and decrease the adverse effects. It has been reported that formulations of vesicular system lead to better result in the treatment of acne compared to conventional system by releasing the drug on targets in skin appendages and these systems are more suitable for lipophilic drug [14, 17].  Table 5 enlists the examples of various liposomal formulations used in acne.

#### 5.1.2. Disadvantage of Liposomal Formulation

The major disadvantage of liposomal formulation is related to its stability aspect. The stability issue of liposomes remains an area, which is surrounded by a number of problems; due to the formation of ice crystals in liposomes, the subsequent instability of bilayers leads to the leakage of entrapped material. The physical instability is also faced by liposomes. The oxidation of cholesterol and phospholipids leads to the formulation instability. Chemical instability indicates the hydrolysis and oxidation of lipids. The destabilization of liposomes is due to the lipid exchange between the liposomes and HDLs [61].

### 5.2. Niosomes

Niosomes are unilamellar or multilamellar vesicles wherein an aqueous phase is encapsulated in highly ordered bilayer made up of nonionic surfactant [62]. They are nonionic surfactant vesicles by which skin penetration and accumulation are increased in the superficial skin strata [63].

#### 5.2.1. Advantage of Niosomal Formulation

Niosomes are one of the promising drug delivery systems in the treatment of skin disorders. When applied topically, niosomes can enhance the residence time of drug in the stratum corneum and epidermis, while systemic absorption of the drug can be reduced [89–92]. They also increase the horny layer properties by reducing transepidermal water loss and increasing the smoothness via replenishing lost skin lipids [63, 93, 94].  Table 5 describes the niosome formulations and their outcomes for treatment of acne. Both niosomes and liposomes are equiactive in drug delivery potential and both increases the drug efficacy as compared with that of free-drug. Niosomes are preferred over liposomes because the former exhibit high chemical stability and economy. One of the reasons for preparing niosomes is that they assume higher chemical stability of the surfactants than that of phospholipids, which are used in the preparation of liposomes. Due to the presence of ester bond, phospholipids are easily hydrolysed [95].

#### 5.2.2. Disadvantages of Niosomes

Although niosomes are superior to liposomes, they have some stability problems associated with them such as physical stability of fusion, aggregation, sedimentation, and leakage on storage. The major issue is the hydrolysis of encapsulated drugs which limits the shelf life of the dispersion in niosomes [96].

### 5.3. Microsponges

Microsponges are uniform, spherical, and porous polymeric delivery system having size range of 5–300 *μ*m [97, 98]. They represent a myriad of interconnecting voids within a noncollapsible structure with a large porous surface loaded with the active agent [54]. It is a microscopic sphere capable of absorbing skin secretions, therefore reducing the oiliness of the skin.

#### 5.3.1. Advantage of Microsponge

Topical agents are a mainstay in cosmetics and the treatment of dermatological disorders. Microsponge delivery system when applied to the skin, the release of drug can be controlled through diffusion or other variety of triggers, including rubbing, moisture, pH, friction, or ambient skin temperature [99]. Controlled release of drug from a delivery system to the skin could reduce the side effect while reducing percutaneous absorption. Microsponges are capable of absorbing skin secretions, therefore reducing oiliness and shine from the skin. Microsponge polymers possess the ability to load a wide range of actives providing the benefits of enhanced product efficacy, mildness, tolerability, and extended wear to a wide range of skin therapies [100, 101]. As compared to liposomes, which suffer from lower payload, difficulty in formulation, limited chemical stability, and microbial instability, the microsponge system in contrast is stable over range of pH 1 to 11 and temperature up to 130°C; compatible with most vehicles and ingredients, self-sterilizing as average pore size is 0.25 *μ*m where bacteria cannot penetrate, higher payload (50 to 60%), still free flowing, and cost effective [9, 10]. One of the most suitable examples is the microsponge of benzoyl peroxide, for topical delivery which maintained efficacy with decreased skin irritation and sensitization [102].  Table 5 shows the various microsponge delivery systems used for treatment of acne.

### 5.4. Microemulsion and Nanoemulsion

Microemulsions are transparent dispersions of oil and water having droplet size of 100 nm in diameter stabilized by an interfacial film of surfactant and cosurfactant molecules [103, 104]. Surfactant and cosurfactant are used to decrease the interfacial tension between oil and water phase [47].

#### 5.4.1. Advantage of Microemulsion

(Co-)surfactant acts as penetration and occlusivity enhancer that improves skin penetration to variable degrees [105]. In microemulsion, active agents are solubilised and thus they are available for quick penetration into the skin. Nanoemulsions (oil in water or water in oil formulation) are characterized by the dispersion of very small sized droplets when mixed. They are appropriate carrier for the transport of lipophilic compounds into the skin and are considered as ideal vehicle for use in acne. This increases the penetration of active component inside the lipophilic environment of the pilosebaceous unit. They also produce additional therapeutic effects like increased skin hydration and viscoelasticity.  Table 5 highlights the various microemulsion formulations used in acne.

### 5.5. Microspheres

It is well said “poor adherence is directly linked to poor treatment results and patient dissatisfaction” [106]. Irritation commonly associated with topical therapies is one of the most significant factors contributing to lack of adherence and therefore therapeutic withdrawal. Microspheres are small spherical shaped particles made of biodegradable polymer and is filled with drug substance that is dispersed homogenously throughout the core and these spheres when degraded, releases the drug for desired time. These microspheres act as a reservoir system for the active agent [40, 54]. Microencapsulation technique is mainly used for the preparation of the microspheres which provide fine coating of inert, natural, and synthetic polymeric materials deposited around solid and liquid micronized particles [42].

#### 5.5.1. Advantage of Microspheres

Microspheres when administered to the skin, the amount of free drug in the formulation penetrates into the epidermis and is compensated by drug release from the microspheres. This system offers sustained drug delivery without overloading the epidermis or resulting an increase in the transdermal penetration [40, 42].  Table 5 enlists the microsphere formulations of drugs used in treatment of acne. Microsphere formulation of topical tretinoin and BPO (benzoyl peroxide) currently on the market has demonstrated good efficacy and tolerability and is expected to encourage adherence and long-term therapeutic benefit. Microsphere encapsulation protects the stability of drugs and makes them photostable. Furthermore, microspheres appear to absorb sebum from the skin's surface, reducing oiliness, which is a common complaint among acne patients [107].

### 5.6. Solid Lipid Nanoparticles (SLNs)

Solid lipid nanoparticles (SLN) were introduced in the year 1991 and they embody an alternative carrier system to tradition colloidal carriers such as emulsions, liposomes, and polymeric carriers. Solid lipid nanoparticles (SLNs) are particles made from solid lipids with a mean diameter between approximately 50 and 1000 nm, which are normally stabilized by lecithin [108, 109]. The reasons for the ever-increasing applications of lipid based system are manyfold and include the following: lipids enhance the oral bioavailability and reduce plasma profile variability, better characterization of lipoid excipients, and an improved ability to address the key issues of technology transfer and manufacture scale-up.

#### 5.6.1. Advantage of SLN

The release rate of the drug from SLNs depends on the presence of the drug in the solid lipid matrix. If the drug is localized only in the outer shell, burst release will be obtained and not controlled release. If the drug is homogeneously distributed within the lipid matrix, however, controlled release can be achieved [110, 111].  Table 5 enlists the SLN-based formulations for acne.

#### 5.6.2. Disadvantages of SLN

Some of the parameters, which hinder the use of SLN, are particle growth, unpredictable gelation tendency, and unexpected dynamics of polymeric transitions.

### 5.7. Hydrogel

Hydrogels are the network of polymer chains that are water-insoluble, and sometimes they are found as a colloidal gel in which water is the dispersion medium. Hydrogels are superabsorbent natural or synthetic polymers [86, 112].

#### 5.7.1. Advantage of Hydrogel

Hydrogels are three dimensional, hydrophilic networks that hold large amount of water or biological fluids, similar to biological tissues. Because of this unique property, hydrogels show good biomedical applications. By tuning, the physicochemical properties of the hydrogels suitable modulated drug delivery system are generated [112].  Table 5 explains the objective of hydrogel formulation of triclosan and tretinoin.

### 5.8. Aerosol Foams

The products packed under pressure and that contain therapeutic active ingredients, which are released upon activation of an appropriate valve system, are called aerosols. These foams are suitable for topical application to the skin and local application into the nose, lungs, and mouth. Aerosol foams are one of the novel drug delivery system used in treatment of acne vulgaris. Foams are preferred for application on large hairy surfaces like the chest, back, and in the face as cleansers due to easiness of application [113, 114].

#### 5.8.1. Advantage of Aerosol Foams

The physicochemical characteristics of vehicle base of the aerosol foam are the same as those of the conventional vehicles like creams, lotions, and gels, having a liquid or semisolid consistency, but aerosol foam maintains desirable properties such as moisturizing fast drying effects or higher drug bioavailability. Gas pressurized system is used to dispensed the aerosol foam [113, 114].  Table 5 explains the objective of aerosol formulation of salicylic acid and benzoyl peroxide.

### 5.9. Fullerenes

Like hollow sphere, fullerenes are molecules composed of carbon. It is reported that when fullerenes are exposed to the skin, they migrate through the skin intercellularly, as opposed to moving through cells. Therefore, a fullerene could be used to “trap” active compounds and then release them into the epidermis once they are applied on the skin. Moreover, fullerenes, themselves, are thought to be potentially potent antioxidants. Literature on fullerenes proved that they can be tolerated and can hold substantial promise in dermatologic and cosmetic applications.

#### 5.9.1. Advantage of Fullerenes

Fullerenes are an excellent antioxidant and a safe material for the suppression of acne vulgaris. This occurred by the inhibition of lipid peroxidation because of fullerene's antioxidant activity and the suppression of sebum production without the production of any side effects. Thus, fullerenes can serve as novel carriers for treatment of acne.

### 5.10. Lipospheres

Lipospheres are lipid-based encapsulation system, used for topical drug delivery of various medicaments. Lipospheres consists of water dispersible solid microparticles, which have diameter ranging from 0.1 to 100 *μ*m. In liposphere, solid hydrophobic fat core is stabilized by a layer of phospholipid molecules embedded in their surfaces, which are a potential group of penetration enhancers [115–119].

#### 5.10.1. Advantage of Lipospheres

Better physical stability, high dispersability in aqueous medium and prolonged release of various types of drugs including anti-inflammatory compounds, local anesthetics, antibiotics, and anticancer agents are possible using this type of system [120–122].

### 5.11. Polymers

Polymers are large molecules, which consist of repeating structural units of monomers connected by chemical covalent bonds. In dermatology, the new acrylic acid polymer turns into gel in presence of water by trapping water into microcells. A stable gel-like formulation containing hydrophilic compound as solution and lipophilic compound in the form of suspension is easy to use, and it releases the active compound after single application. For example, an antiacne formulation that combines clindamycin (1%) and benzoyl peroxide (5%) utilizes this novel polymer-based gel technology and provides excellent tolerability and efficacy.

Despite the availability of numerous effective medical therapies for acne vulgaris, issues of safety, compliance, and less than ideal efficacy help drive the search for alternative treatments for this exceedingly common clinical problem. Recently, scientists have developed effective vaccine for *P. acnes*-associated inflammatory acne, consisting of a cell wall-anchored sialidase of *P. acnes* or killed-whole organism of *P. acnes* [123, 124]. They also hope to develop a future bacterial therapy for overcoming problems seen with the continuous use of antibiotics such as a building up a bacteria resistance. These scientists of the 21st century are convinced that acne is not due to dirt and that scrubbing skin can lead to worse problems. Therefore, in the future, it is possible to explore the use of micro- and nanocarrier-based drug delivery systems in advanced form with increase in effectiveness for treatment of acne.

### 5.12. Nanostructured Lipid Carriers

Nanostructured lipid carriers are smarter second-generation drug carrier systems having solid matrix at room temperature. This carrier system is usually made up of physiological, biodegradable, and biocompatible lipid materials and surfactants and is accepted by regulatory authorities for application in different drug delivery systems. NLCs exhibit superior advantages over other colloidal carriers like nanoemulsions, polymeric nanoparticles, liposomes, SLN, and so forth and thus they are been explored to more extent in drug delivery.

#### 5.12.1. Advantages of NLCs

The unique set of advantages of NLCs includes enhanced drug loading capacity, prevention of drug expulsion, and more flexibility for modulation of drug release. For example, Zhou, prepared adapalene (a retinoid antiacne drug) loaded nanostructured lipid carriers for topical use. These NLCs were able to accumulate in hair follicles and improve the follicular delivery of adapalene. Thus, NLCs could be promising carriers for topical delivery of antiacne drugs.

### 5.13. Cyclodextrin Based Carriers

Cyclodextrins (CDs) are a family of cyclic oligosaccharides derived from starch containing six (*α*-CD), seven (*β*-CD), eight (*γ*-CD), or more (*α*-1,4)-linked *α*-D-glucopyranose units. They take the shape of a truncated cone or torus instead of a perfect cylinder because of the chair conformation of the glucopyranose units. These versatile, pharmaceutical-material CDs are classified into hydrophilic, hydrophobic, and ionic derivatives [125]. Cyclodextrin complexation is a well known technique for enhancing the solubility and stability of drug, sustaining the release and minimizing the photo degradation of drug. In particular, the focus of investigation involves the combination of vesicular approach with cyclodextrin complexation (dual approach) which would help in increasing the solubility, skin permeation, and deposition and reducing the photodegradation of drugs. Nowadays drug-cyclodextrin-vesicles dual carrier approach for targeting of antiacne agent to skin is used. For example, Kaur et al. prepared isotretinoin-hydroxypropyl-*β*-cyclodextrin (HP-*β*-CD) inclusion complex and encapsulated this complex in elastic liposomes and studied the effect of dual carrier approach on skin targeting [68]. The isotretinoin elastic liposomal formulation possessed great potential for skin targeting, prolonging drug release, reduction of photodegradation, reducing skin irritation, and improving topical delivery.

## 6. Conclusion

Adolescent stage is a complex life cycle characterized by many striking biological, psychological, physical, and social changes. It is a labile stage where most self-esteem development occurs, whereas low self-esteem is associated with anxiety, depression, and increased reports of general psychiatric morbidities. The physical changes of acne may have negative effect on the psychology, self-esteem, and quality of life of adolescents. Although many traditional oral and topical medical agents have been demonstrated to be effective in the treatment of acne, the prevalence of the disease and its frequently resistant nature make the development of alternative therapies highly desirable. There has been significant progress over the past few years, but not all developments can be universally applied. An effective topical formulation must provide stability and enhanced penetration of active ingredients at optimal concentrations for efficacy and it should be acceptable and cheaper and should not add side effects of its own. The encapsulation of antiacne drugs in (vesicular and particulate) carrier delivery systems represents an innovative alternative to minimize the side effects, while preserving their efficacy. They can enhance the dermal and transdermal use and can alter the skin penetration. The penetration rate can increase or decrease depending on the nature of the active agent and the preparation. Improved uptake is often linked with higher efficacy and minimizes the side effect. The capacity of these systems can provide controlled release to improve the drug penetration into skin or even into the pilosebaceous unit. If the concentration of the active pharmaceutical ingredient is adjusted, local tolerability can be improved. Currently, only very few drugs based on microsized or nanosized application systems have been approved for topical use and introduced into the market. Much progress has been made to improve the performance of antiacne care products in recent years. These new formulations based on carrier system provide efficacy, tolerability, compliance, and cosmetic acceptability. In coming future, the use of cyclodextrin based carriers and their delivery system will be more beneficial as it covers dual approach comprising the advantage of both system and lead to development of safe and effective formulation, which would be cost effective, and save time and labor.

## Figures and Tables

**Table 1:** Types of acne.

Sr. no	Type of acne	Features
1	Comedonal (noninflammatory)	*Whitehead* (*closed*): a dilated hair follicle filled with keratin, sebum, and bacteria, with an obstructed opening to the skin. *Blackhead* (*open*): a dilated hair follicle filled with keratin, sebum, and bacteria, with a wide opening to the skin capped with a blackened mass of skin debris.

2	Papulopustular (inflammatory)	*Papule*: small bump less than 5 mm in diameter. *Pustule:* smaller bump with a visible central core of purulent material.

3	Nodular (inflammatory)	*Nodule*: bump greater than 5 mm in diameter.

**Table 2:** Types of acne according to severity.

Sr. no	Type of acne	Features
1	Mild acne	Fewer than 20 comedones or fewer than 15 inflammatory lesions, or total lesion count fewer than 30

2	Moderate acne	20–100 comedones, or 15–50 inflammatory lesions, or total lesion count 30–125

3	Severe acne	More than 5 nodules, or Total inflammatory count greater than 50, or Total lesion count greater than 125

**Table:** (a) Systemic treatment

Antibiotic	Name	Dose	Duration	Drawbacks
Oral antibiotics				
Tetracyclines	Tetracycline, Oxytetracycline	250–500 mg twice daily	4–6 months	Gastrointestinal upset, vaginal candidiasis, decreased compliance
	Doxycycline	50–100 mg twice daily	4–6 months	Gastrointestinal upset, photosensitivity
	Minocycline	50–100 mg twice daily	4–6 months	Vertigo, hyperpigmentation of skin and oral mucosa, expensive
	Lymecycline	150–300 mg daily	4–6 months	
Macrolides	Erythromycin	500 mg twice daily	4–6 months	Gastrointestinal upset, vaginal candidiasis, emergence of resistance of *P. acnes*
	Azithromycin	250 mg three times a week	4–6 months	Gastrointestinal upset

**Table:** (b) Hormonal treatment

Name	Dose	Duration	Drawbacks
Spironolactone	25–100 mg twice daily	6 months	Menstrual irregularities, contraindicated in pregnancy
Prednisone	2.5–5 mg daily	Indefinitely	Adrenal suppression
Dexamethasone	0.125–0.5 mg daily	Indefinitely	Adrenal suppression
Cyproterone acetate/ethinyl estradiol (oral contraceptives)	2 mg/35–50 *μ*g	6 months	Vascular thrombosis, melasma, weight gain
Levonorgestrel/ethinyl estradiol	100 *μ*g/20 *μ*g	6 months	Vascular thrombosis, melasma, weight gain

**Table 4:** Topical conventional delivery system used for acne.

Conventional delivery system	Drug	Side effects	Reference
Lotion	Benzoyl peroxide	Peeling, itching, redness, dryness, burning, and dermatitis	[41]
	Clindamycin	Peeling, itching, redness, dryness	[42]
	Tretinoin	Erythema, scaling, burning	[43]
	Erythromycin	Erythema, scaling, burning	[44]
	Glycolic acid	Itching, rash, pruritus	[45]
	Tretinoin	Itching, rash, pruritus	[45]

Cream	Adapalene	Erythema, scaling, dryness, burning, stinging, irritation, sunburn	[46]
	Tazarotene	Erythema, scaling, burning	[47]
	Azelaic acid	Itching, rash, pruritus	[45]
	Tea oil	Burning, itching, irritation, stinging	[48]
	Clindamycin	Erythema, desquamation	[49]

Gel	Salicylic acid	Erythema, dryness, dermatitis	[41, 50]
	Erythromycin	Dryness, erythema, peeling, dermatitis	[44, 51]
	Benzoyl peroxide	Dryness, erythema, peeling, dermatitis	[51]
	Adapalene	Erythema, scaling, dryness, burning, stinging, irritation	[46]
	Dapsone	Peeling, itching, redness, burning	[52]

Emollient	Sodium sulfacetamide-sulfur	Dryness, irritation, redness, scaling, stinging, or burning	[53]

**Table 5:** Novel carrier-based delivery system for acne.

Drug	Objective	Outcomes	Reference
Liposomal formulation			
Benzoyl peroxide	To improve the antibacterial efficacy of benzoyl peroxide	A significant antibacterial effect in the infundibula against both *P. acne* and Micrococcaceae was observed as compared to the conventional formulation.	[64]
Clindamycin	To improve the stability and penetrability	Increased stability and intradermal penetrability	[65]
Salicylic acid	To reduce associated side effects	Liposomal formulation produced fivefold higher deposition of drug in skin than the corresponding plain drug solution and conventional gel and reduced skin irritation was observed.	[66]
Tretinoin	To improve the stability and the thermodynamic activity	Increased stability and drug retention were achieved.	[67]
Isotretinoin	To increase skin targeting and skin deposition and reduce skin irritation.	Increase skin targeting, drug deposition and decrease skin irritation were observed.	[68]
Lauric acid	To evaluate the antimicrobial activity	Lauric acid loaded liposomes release the drug directly into the bacterial membranes, thereby killing the bacteria effectively.	[14]
Cyproterone acetate	To increase percutaneous absorption	Better penetration was observed	[69]
Finasteride	To increase skin permeation, deposition, and stability of the drug.	Higher deposition of drug in skin, increased permeation and stability were observed.	[70]
Tea oil	To increase skin permeability of drug.	Tea oil liposome disrupted the permeability barrier of cell membrane structures and increased the permeability.	[48]
Methylene blue	To evaluate the efficacy and tolerability of liposomes loaded methylene blue.	Liposomal formulation delivered the methylene blue to sebaceous gland and was effective in treatment of mild-to-moderate acne vulgaris.	[71]

Niosome formulation			
Benzoyl peroxide	To reduce the associated side effects	Niosomal gel improved the skin retention, therapeutic response and considerably reduced the adverse symptoms.	[72]
Tretinoin	To improve skin drug retention of drug and increase photostability.	Niosomal formulation improved the cutaneous or transdermal delivery of a lipophilic tretinoin and increased photostability.	[63]
Erythromycin	To enhance drug retention into skin and improve stability.	Niosomal gel was significantly more stable as compared to plain drug gel and marketed gel and drug retention was increased.	[72]

Microsponge formulation			
Benzoyl peroxide	To reduce skin irritation.	Controlled release and reduced skin irritation	[73]
Tretinoin	To reduce cutaneous side effect	Controlled release of tretinoin with reduced cutaneous side effects.	[74]

Microemulsion formulation			
Tretinoin	To increase skin permeation and skin retention.	Novel microemulsion increases tretinoin penetration through skin and maximum amount of drug retained as compare to plain drug in solution, gel and marketed preparation.	[75, 76]
Retinoic acid	To increase lipophilicity and skin permeability.	The O/W micro emulsions containing a counter ion increased the skin permeability and lipophilicity of drug.	[77]

Microsphere formulation			
Benzoyl peroxide	To reduce skin irritation on topical treatment.	Cream containing microspheres of benzoyl peroxide offered favorable efficacy with a very low potential for irritation.	[78]
Tretinoin	To reduce cutaneous irritation, including erythema, peeling, dryness, burning, and itching.	Microsphere formulation reduced local side effects and sustained release was achieved.	[79]
All trans retinoic acid	To control the release of drug.	Controlled release of drug was produced by encapsulation of drug into the microsphere.	[80]

Solid lipid nanoparticles formulation			
Tretinoin	To evaluate the potential of a lipophilic drug with respect to primary skin irritation, in vitro occlusivity and skin permeation.	Lesser skin irritancy, greater skin tolerance, occlusivity, slow drug release, and increased permeability were observed with the developed tretinoin loaded SLN-based gels more than the commercial product.	[81]
Isotretinoin	To evaluate skin penetration	SLN loaded with isotretinoin significantly increased the accumulative uptake of drug into the skin and enhanced the skin permeation.	[82]
All trans retinoic acid	To produce comedolytic effect and reduce skin irritation.	SLN produced comedolytic effects and epidermal thickening with reduced skin irritation.	[73]
Sphingosome	To increase skin permeation of drug.	Sphingosome SLN enhanced the permeation of the drug through the skin to acne lesion.	[83]
Cyproterone acetate (CPA)	To reduce side effect and improve skin penetration and absorption.	CPA attached to SLN increased skin penetration at least four-fold over the uptake from cream and nanoemulsion. Incorporation of drug into the lipid matrix of NLC resulted in a 2 to 3 fold increase in CPA absorption.	[84]
Triclosan	To increase stability, skin retention and permeability.	Triclosan nanoparticle increased the stability and showed higher retention and permeability than conventional cream formulation.	[85]

Hydrogel formulation			
Triclosan	To increase the permeability through skin.	Triclosan permeability was increased by using transcutol as a permeation enhancer.	[86]
Tretinoin	To increase release permeation and reduce skin irritation of tretinoin.	The complexation of tretinoin with dimethyl-*β*-cyclodextrin overcome the drug's low water solubility thereby increasing drug release and enhanced the drug permeation by promoting skin absorption and alleviate drug inducing local irritation.	[87]

Aerosol foams formulation			
Juniper oil	To reduce the volatility and maintain antibacterial activity.	Juniper oil solid lipid microparticles substantially maintain the oil loaded inside their lipidic structure, reducing its volatility and retaining its antibacterial activity.	[88]
